# Transforming growth factor β 869C/T and interleukin 6 -174G/C polymorphisms relate to the severity and progression of bone-erosive damage detected by ultrasound in rheumatoid arthritis

**DOI:** 10.1186/ar3396

**Published:** 2011-07-08

**Authors:** Fulvia Ceccarelli, Carlo Perricone, Martina Fabris, Cristiano Alessandri, Annamaria Iagnocco, Cinzia Fabro, Elena Pontarini, Salvatore De Vita, Guido Valesini

**Affiliations:** 1 Reumatologia, Dipartimento di Medicina Interna e Specialità Mediche, Sapienza Università di Roma, viale del Policlinico 155, I-00161 Rome, Italy; 2 Cattedra di Reumatologia, DPMSC, Università degli Studi di Udine, Via Palladio, 8 Palazzo Florio, I-33100, Udine, Italy

## Abstract

**Introduction:**

Single nucleotide polymorphisms (SNPs) of transforming growth factor β (TGF-β) and IL-6 genes (respectively, 869C/T and -174G/C) have been associated with radiographic severity of bone-erosive damage in patients with rheumatoid arthritis (RA). Musculoskeletal ultrasound (US) is more sensitive than radiography in detecting bone erosion. We analyzed the association between TGF-β 869C/T and IL-6 -174G/C SNPs and bone-erosive damage, evaluated by US, in a cohort of patients with severely active RA.

**Methods:**

Seventy-seven patients were enrolled before beginning anti-TNF treatment. Disease activity was measured using the disease activity score in 28 joints, and the clinical response was evaluated according to the European League Against Rheumatism response criteria. Rheumatoid factor (RF) and anticitrullinated protein/peptide antibodies (ACPAs) were detected. The 869C/T TGF-β and -174G/C IL-6 SNPs were analyzed by PCR amplification. US was performed to assess the bone surfaces of metacarpophalengeal (MCP), proximal interphalangeal (PIP) and metatarsophalangeal (MTP) joints by obtaining multiplanar scans. According to the number of erosions per joint, a semiquantitative score ranging from 0 to 3 was calculated in each anatomical site to obtain a MCP total erosion score (TES), a PIP TES and a MTP TES, all ranging from 0 to 30, and a global patient TES calculated as the sum of these scores (range, 0 to 90).

**Results:**

Patients carrying the TGF-β 869TT genotype showed a statistically significant lower MTP TES than those with the CC or CT genotype (mean MTP TES ± standard deviation for 869TT 6.3 ± 5.7 vs. 869CC/CT 11.7 ± 7.8; *P *= 0.011). Interestingly, patients with the TT genotype showed dichotomous behavior that was dependent on autoantibody status. In the presence of ACPAs and/or RF, the TT genotype was associated with lower erosion scores at all anatomical sites compared with the CC and CT genotypes. Conversely, the same 869TT patients showed higher erosion scores in the absence of ACPAs or RF.

**Conclusions:**

In RA patients, TGF-β 869C/T SNPs could influence the bone-erosive damage as evaluated by US. The serological autoantibody status (ACPAs and RF) can modulate this interaction.

## Introduction

Rheumatoid arthritis (RA) is a chronic, systemic inflammatory disease affecting primarily the joints. Its prevalence is approximately 0.5% to 1% in the industrialized countries [1]. The genetic background of patients with RA is responsible for at least part of the disease susceptibility and phenotype as demonstrated by twin and family studies. The human leukocyte antigen (HLA)-DRB1 shared epitope (SE) locus is strongly associated with the disease, accounting for approximately one-third of the genetic component of RA susceptibility [2]. Thus, other non-HLA genes may play a role in RA disease development, and previous research has focused on genes encoding for cytokines in key pathogenetic pathways. Transforming growth factor β (TGF-β) is a modulator of the immune response in RA. The effects exerted by this cytokine are midway between pro- and anti-inflammatory, depending on several, mostly unveiled, factors. TGF-β promotes the differentiation of leukocytes while inhibiting the proliferation of T lymphocytes and the activation of monocytes and/or macrophages [3]. Recently, three independent study groups simultaneously discovered that if TGF-β is displaced in an inflammatory milieu, it might act synergistically with IL-6 to induce the differentiation of naive T cells into Th17 cells [4-6]. This cell lineage is characterized by the production of IL-17, a proinflammatory cytokine associated with joint inflammation, osteoclastogenesis and the development of bone-erosive damage [7]. IL-6 is one of the main determinants of inflammation in RA. Indeed, it promotes the synthesis of acute phase reactants by the liver, can regulate inflammatory and/or immune pathways and modulate bone metabolism and endocrine function [8].

Single nucleotide polymorphisms (SNPs) of the TGF-β and IL-6 genes (869C/T and -174G/C, respectively) have been associated with RA susceptibility and radiographic severity of bone-erosive damage [9-13]. Nowadays, conventional radiography is considered a well-established imaging technique for identifying progressive joint damage. However, musculoskeletal ultrasound (US) is more sensitive in the detection of soft-tissue lesions and bone erosion [14].

The first aim of our study was to analyze whether TGF-β 869C/T and IL-6 -174G/C are associated with bone-erosive damage on the basis of US evaluation in a cohort of RA patients starting anti-TNF treatment. A secondary aim was to assess whether these SNPs could influence US bone erosion progression after six months of anti-TNF therapy.

## Materials and methods

Seventy-seven patients with established RA diagnosed according to the 1987 revised American College of Rheumatology (ACR) criteria [15], were enrolled at the Rheumatology Unit of Sapienza University of Rome. Patients' diagnoses were confirmed according to the recently published European League Against Rheumatism (EULAR)/ACR 2010 criteria [16]. The patients started anti-TNF therapy with either subcutaneous adalimumab 40 mg every other week (*n *= 12) (Humira; Abbott Laboratories Ltd, Vanwall Business Park, Vanwall Road, Maidenhead, Berkshire, UK) or subcutaneous etanercept 50 mg once per week (*n *= 65) (Enbrel; Wyeth Europa Ltd., Huntercombe Lane South, Taplow, Maidenhead, Berkshire, UK) for severely active disease refractory to conventional therapy with disease-modifying antirheumatic drugs (DMARDs). The patients were studied before anti-TNF treatment was started (baseline = T0) and at three and six months after initiation of anti-TNF therapy (T3 and T6, respectively). DMARD and glucocorticoid doses were maintained at a stable level during follow-up. The local ethical committee approved the study, which was performed according to the Declaration of Helsinki criteria, and all patients provided their written informed consent for participation in the study.

### Clinical evaluation

All patients were evaluated by the same rheumatologist (FC). Data were collected and entered into a standardized, computerized, electronically filled-in form as previously described [17]. Data included patient demographics, date of diagnosis, comorbidities and previous and concomitant medications. The clinical evaluation included a count of swollen and tender joints and the patient's and physician's global disease assessment based on a visual analogue scale (VAS; range, 0 to 100 mm). Disease activity was measured according to the disease activity score in 28 joints (DAS28), and the clinical response was evaluated according to the EULAR response criteria [18]. The patients were asked to fill in the Health Assessment Questionnaire (HAQ) [19].

### Laboratory analysis

Blood samples were obtained from all subjects, and genomic DNA and sera were collected using standard protocols and stored at -70°C until use. Rheumatoid factor (RF) (normal value < 17 IU/mL) (Behring, Marburg, Germany) and anticitrullinated protein/peptide antibodies (ACPAs) (normal value < 5 IU/mL) (Axis-Shield plc, Dundee, UK) were detected by ELISA according to the manufacturers' instructions. For each patient, we also measured the erythrocyte sedimentation rate (normal value < 20 mm/hour) by using the Westergen method, as well as the C-reactive protein level (normal value < 5 mg/dL).

### Genotyping

DNA was extracted from ethylenediaminetetraacetic acid-treated peripheral blood using an automated methodology (Maxwell 16; Promega, Madison, WI, USA) and dedicated kits (Maxwell 16 Blood DNA Purification Kit; Promega). The 869C/T SNP was analyzed by PCR amplification and digestion with a site-specific restriction enzyme in accordance with previously reported methods [13]. The forward and reverse primers were 5'-TTCCCTCGAGGCCCTCCTA-3' and 5'-GCCGCAGCTTGGACAGGATC-3', and the PCR amplification protocol was composed of 35 cycles comprising three steps each: 75 seconds at 96°C, 75 seconds at 62°C and 75 seconds at 73°C. PCR products were digested with *Msp*A1I (New England BioLabs, Ipswich, MA, US) and run on a 3% ethidium bromide-stained agarose gel.

The -174G/C IL-6 promoter SNP was analyzed by PCR amplification and digestion with a site-specific restriction enzyme using previously reported methods [20]. The forward and reverse primers were 5'-TGACTTCAGCTTTACTCTTGT-3' and 5'-CTGATTGGAAACCTTATTAAG-3', and the PCR amplification protocol was composed of 39 cycles comprising three steps each: one minute at 95°C, one minute at 55°C and one minute at 72°C. PCR products were digested with *Nla*III (New England BioLabs, Ipswich, MA, US) and run on a 3.5% ethidium bromide-stained agarose gel.

### Musculoskeletal ultrasound assessment

US imaging was performed by using a MyLab70 XVG machine (Esaote S.p.A., Florence, Italy) equipped with a 6- to 18-MHz linear probe. By using a fixed 18-MHz frequency, bone surfaces of metacarpophalangeal (MCP), proximal interphalangeal (PIP) and metatarsophalangeal (MTP) joints were studied on multiplanar scans in accordance with the EULAR US guidelines [21]. We chose these joints because previous reports have shown that bone erosion in RA may preferentially develop in the small joints of the feet and hands at early stages of the disease [22]. Gel was applied to the skin to provide an acoustic interface. The first through the fifth MCP joints of both hands, the first interphalangeal and the second through fifth PIP joints of both hands, and the first through fifth MTP joints of both feet were scanned. Each joint was scanned in both the longitudinal and transverse planes from the medial to lateral sides on both volar and dorsal aspects to enable maximum coverage of the joint surface area. To increase the acoustic window or access of the transducer between the joints of specific fingers, the fingers were splayed and then made into a fist. The scans were obtained independently on the same day by two rheumatologists (FC and CP) trained and experienced in sonography. Each sonographer was blinded to the sonographic findings of the other observer, but not to the diagnosis. Bone erosion was assessed according to the Outcome Measures in Rheumatoid Arthritis Clinical Trials (OMERACT) definitions [23]. Based on the number of erosions per joint, a semiquantitative score ranging from 0 to 3 was applied (grade 0 = no erosion, grade 1 = one erosion, grade 2 = two erosions and grade 3 = at least three erosions). The sum of the scores per joint in articulations from the same anatomical site gave the MCP, PIP and MTP total erosion score (TES) (MCP TES, PIP TES and MTP TES, respectively; range, 0 to 30). A global patient TES was obtained by calculating the sum of these scores (range, 0 to 90). All scores were the means ± standard deviations (SDs) of the scores obtained by the independent evaluation of the two sonographers.

### Statistical analysis

The statistical analyses were performed using the Statistical Package for Social Sciences version 13.0 software (SPSS, Inc., Chicago, IL, USA). Comparisons of gene and genotype frequencies between the groups were performed by using contingency tables and Pearson's χ^2 ^test. Corrections were made where necessary for the sample size (Fisher's exact test). Normally distributed variables were summarized using the means (± SD), and non-normally distributed variables were expressed as medians and interquartile ranges. The comparisons between nonparametric variables were performed using the Mann-Whitney *U *test. Kruskal-Wallis one-way analysis of variance was applied to evaluate the comparisons between multiple groups. The Bonferroni correction was adopted (*P*_c_). Pearson's and Spearman's tests were used to perform the correlation analysis. Interobserver reproducibility was determined using κ statistics, and κ values were evaluated according to the method of Landis and Koch [24]. All the *P *values were two-tailed, and *P *< 0.05 was considered significant.

## Results

### Demographics, clinical and laboratory features

The demographics and the clinical and laboratory features of the 77 RA patients are given in Table 1. At the time of study entry, our patients showed moderately to severely active disease (mean DAS28 (± SD) 5.2 ± 1.2) and moderate functional disability (mean HAQ (± SD) 1.26 ± 0.8). Fifty-eight patients (75.3%) were ACPA-positive, while 61 (79.2%) were RF-positive. Concerning concomitant treatment for RA, 48 patients (62.3%) were taking corticosteroids and 53 (68.8%) were being treated with at least one DMARD.

**Table 1:** Clinical, laboratory and ultrasonographic characteristics of the 77 enrolled RA patients at study entry^a^

Characteristics	Data
Demographics	
Males/females, *n *(%)	10 (13)/67 (87)
Mean age, years (± SD)	55.9 ± 14.3
Mean disease duration, months (± SD)	119.2 ± 93.6
Caucasian, *n *(%)	67 (87)
Hispanic, *n *(%)	9 (11.7)
African, *n *(%)	1 (1.3)
Laboratory results	
RF-positive, *n *(%)	61 (79.2)
ACPA-positive, *n *(%)	58 (75.3)
Mean ESR, mm/hour (± SD)	31.8 ± 24.7
Clinical status (± SD)	
Mean DAS28 score	5.2 ± 1.2
Mean HAQ score	1.26 ± 0.8
Concomitant treatment	
Corticosteroids, *n *(%)	48 (62.3)
DMARDs, *n *(%)	
Methotrexate	37 (48.0)
Hydroxychloroquine	16 (20.8)
Salazopyrin	15 (19.5)
Leflunomide	9 (11.7)
Mean US results (± SD)	
Overall patient TES	33.4 ± 21.9
MCP TES	13.2 ± 8.1
PIP TES	9.7 ± 8.1
MTP TES	10.4 ± 8

^a^ACPA: anticitrullinated protein/peptide antibody; DAS28: disease activity score in 28 joints; DMARD: disease-modifying antirheumatic drug; ESR: erythrocyte sedimentation rate; HAQ: Health Assessment Questionnaire; MCP: metacarpophalangeal; MTP: metatarsophalangeal; PIP: proximal interphalangeal; RA: rheumatoid arthritis; RF: rheumatoid factor; SD: standard deviation; TES: total erosion score; US: musculoskeletal ultrasound.

### Ultrasonographic evaluation

All the evaluated patients showed the presence of erosions (patient TES range, 2 to 90). The mean US erosion scores at baseline (T0) are given in Table 1. MCP joints showed a significantly higher number of erosions compared with the PIP and the MTP joints (*P *= 0.005 and *P *= 0.03, respectively). Considering the erosive damage according to ACPA status, autoantibody-positive patients showed higher patient TES, although these scores were not statistically significant, compared with the ACPA-negative patients (mean ± SD 36.7 ± 23.4 vs. 24.4 ± 17.7; *P *= NS). Analogously, RF-positive patients showed higher patient TES compared with RF-negative patients (mean ± SD 35.6 ± 23.2 vs. 28.1 ± 16.4; *P *= NS). Mean patient TES increased at T3 (mean ± SD 40.3 ± 22.8) and at T6 (mean ± SD 40.5 ± 22.4) (*P *= NS for both comparisons) (see Table 2).

**Table 2:** Modification of mean DAS28 and TES of the 77 RA patients and patient response percentages according to EULAR criteria at baseline (T0) and after three months (T3) and six months (T6) of anti-TNF therapy^a^

Outcome measure	T0	T3	T6	*P *value
Mean DAS28 (± SD)	5.2 ± 1.2	3.9 ± 1.6	3.8 ± 1.4	T0 vs. T3 < 0.0001
EULAR response	-			-
Good (%)		25	31.8	
Moderate (%)		38.5	25	
None (%)		36.6	43.2	
Mean overall patient TES (± SD)	33.4 ± 21.9	40.3 ± 22.8	40.5 ± 22.4	NS

^a^DAS28: disease activity score in 28 joints; EULAR: European League Against Rheumatism; NS: not significant; PIP: proximal interphalangeal; RA: rheumatoid arthritis; TES: total erosion score; TNF, tumor necrosis factor.

Patients showed a significant DAS28 reduction at T3 (*P *< 0.0001) and remained substantially stable at T6. According to the EULAR criteria, a good or moderate clinical response was achieved in 63.5% of patients at T3 and in 56.8% of patients at T6 (Table 2). At baseline, patient DAS28 and TES showed a positive correlation (*P *= 0.01). Changes in DAS28 did not correlate with changes in TES at T3 or T6.

### Association of bone-erosive damage with TGF-β 869C/T SNP

Twenty-three patients (29.8%) had the TGF-β 869CC genotype, 34 (44.2%) had the TGF-β 869CT genotype and the remaining 20 (26%) had the TGF-β 869TT genotype. After subgrouping the patients according to genotype, no significant differences were observed among the three groups of patients at baseline with regard to mean age, disease duration, ACPA or RF status, disease activity or disability (Table 3).

**Table 3:** Characteristics of the 77 RA patients stratified by TGF-β 869C/T SNPs at baseline (T0) and after three months (T3) and six months (T6) of anti-TNF therapy^a^

Characteristics	T0	T3	T6	*P *value
Mean age, years (± SD)	55.9 ± 14.3	57.1 ± 13.9	56.7 ± 14.1	NS
Mean disease duration, months (± SD)	118.8 ± 93.6	116.4 ± 94.8	118.8 ± 94.8	NS
RF-positive, *n *(%)	18 (78.2)	29 (85.2)	14 (70)	NS
ACPA-positive, *n *(%)	16 (69.5)	28 (82.3)	14 (70)	NS
Mean DAS28 (± SD)	5.3 ± 1.1	5.2 ± 1.1	4.8 ± 1.2	NS
Mean HAQ score (± SD)	1.2 ± 0.7	1.34 ± 0.8	1.28 ± 0.8	NS
Mean overall patient TES (± SD)	34.1 ± 22.1	34.7 ± 24.4	29.5 ± 16.8	NS
Mean MCP TES (± SD)	14.7 ± 8.8	12.6 ± 8.6	12.4 ± 6.2	NS
Mean PIP TES (± SD)	8 ± 7.1	10.3 ± 8.9	10.6 ± 7.8	NS
Mean MTP TES (± SD)	11.3 ± 9	11.7 ± 7.8	6.3 ± 5.7	*P

^a^ACPA: anticitrullinated protein/peptide antibody; DAS28: disease activity score in 28 joints; HAQ: Health Assessment Questionnaire; MCP: metacarpophalangeal; MTP: metatarsophalangeal; PIP: proximal interphalangeal; RA: rheumatoid arthritis; RF: rheumatoid factor; TES: total erosion score; TGF-β: transforming growth factor β. *P < 0.05, CC vs. TT; *P *= 0.01, CT vs. TT.

Interestingly, MTP TES was statistically significantly different between TT genotype patients and those with the CC or CT genotype (mean ± SD MTP TES 869TT: 6.3 ± 5.7 vs. 869CC/CT: 11.7 ± 7.8; *P *= 0.011) (Table 3). The same results were observed when correction for disease duration was performed.

To determine whether the effect of TGF-β SNP on bone-erosive damage could have been influenced by ACPA and RF status, a comparison between autoantibody-positive and autoantibody-negative patients was performed (Table 4). Patients with the 869TT genotype (T allele) showed dichotomous behavior depending on autoantibody status. In the presence of ACPA, these patients showed a trend toward lower erosion scores at all anatomical sites studied compared with ACPA-positive patients with the CC or CT genotype (C allele). MTP TES was statistically significantly different between ACPA-positive patients with the T allele and those with the C allele (CC vs. TT *P*_c _< 0.01 and CT vs. TT *P*_c _< 0.05) (Table 4). Similarly, MTP TES was statistically significantly lower in RF-positive patients with the T allele compared with those with the C allele (CC vs. CT *P*_c _< 0.05 and CT vs. TT *P*_c _< 0.05, respectively). Conversely, in seronegative patients, a trend toward higher erosion scores was observed for patients with the T allele compared with those with the C allele. RF-negative patients with the TT genotype showed significantly higher PIP TES compared with those with the CC genotype (*P*_c _< 0.05).

**Table 4:** Bone-erosive damage assessed on the basis of US scores across TGF-β 869C/T SNPs according to ACPA and RF status^a^

Patient status	CC	CT	TT	*P *value
ACPA-positive				
Mean overall patient TES (± SD)	39.5 ± 21.4	39.4 ± 26.3	23.7 ± 13.7	NS
Mean MCP TES (± SD)	16.7 ± 8.7	14.2 ± 9.1	11.2 ± 5.1	NS
Mean PIP TES (± SD)	9.4 ± 11.9	11.9 ± 9.8	7.7 ± 6.7	NS
Mean MTP TES (± SD)	13.3 ± 8.8	13.1 ± 8.3	4.75 ± 4.2	*P *= 0.008, *P*_c _< 0.01; CC vs. TT
				*P *= 0.01, *P*_c _< 0.05; CT vs. TT
ACPA-negative				
Mean overall patient TES (± SD)	24 ± 21.9	22 ± 11.1	38 ± 17.1	NS
Mean MCP TES (± SD)	11.5 ± 8.4	7.1 ± 3.6	14 ± 4.7	*P *< 0.05, *P*_c _= NS; CT vs. TT
Mean PIP TES (± SD)	5.6 ± 5.3	6.1 ± 5.2	14.2 ± 7.8	*P *< 0.05, *P*_c _= NS; CC vs. TT
Mean MTP TES (± SD)	6.8 ± 9.4	8.6 ± 4.8	9.8 ± 8.3	NS
RF-positive				
Mean overall patient TES (± SD)	38 ± 22.4	37.6 ± 25.7	23.7 ± 13.7	NS
Mean MCP TES (± SD)	16.4 ± 8.9	13.8 ± 8.9	11.2 ± 5	NS
Mean PIP TES (± SD)	8.6 ± 7.6	11 ± 9.6	7.7 ± 6.7	NS
Mean MTP TES (± SD)	13 ± 9.1	12.7 ± 8.1	4.7 ± 4.2	*P *= 0.01, *P*_c _< 0.05; CC vs. CT
				*P *= 0.01, *P*_c _< 0.05; CT vs. TT
RF-negative				
Mean overall patient TES (± SD)	16.5 ± 8.7	27.4 ± 16.5	38 ± 17.1	*P *= 0.03, *P*_c _= NS; CC vs. TT
Mean MCP TES (± SD)	7.5 ± 3.3	8.8 ± 6	14 ± 4.7	*P *= 0.03, *P*_c _= NS; CC vs. TT
Mean PIP TES (± SD)	5 ± 3.5	9 ± 6.8	14.2 ± 7.8	*P *= 0.03, *P*_c _< 0.05; CC vs. TT
Mean MTP TES (± SD)	4 ± 3.5	9.6 ± 8.3	9.8 ± 8.3	NS

^a^ACPA: anticitrullinated protein/peptide antibody; MCP: metacarpophalangeal; MTP: metatarsophalangeal; NS: not significant; PIP: proximal interphalangeal; RF: rheumatoid factor; TES: total erosion score; TGF-β: transforming growth factor β; US: musculoskeletal ultrasound. The comparisons were performed between genotypes (CC vs. CT vs. TT groups) in ACPA-positive and ACPA-negative patients and in RF-positive and RF-negative populations, respectively, using Kruskal-Wallis one-way analysis of variance. *P*_c_: *P *values with the Bonferroni correction.

**Figure 1:**
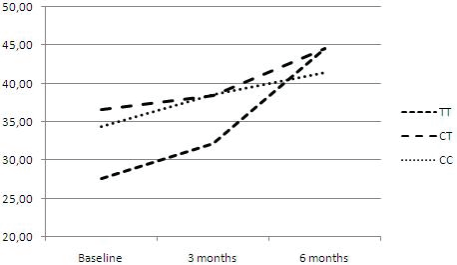
**Representation of changes in US score according to TGF-β 869C/T genotype after three and six months of anti-TNF therapy**.

We also assessed whether the TGF-β 869C/T SNP could have influenced US bone erosion progression during six months of anti-TNF therapy. As shown in Figure 1, after stratifying for TGF-β genotypes, there were no statistically significant differences in US-identified progression of patient TES at T3 or T6.

### Association of bone-erosive damage with IL-6 -174G/C

Forty-eight patients (62.3%) had the IL-6 -174GG genotype, 24 patients (31.2%) had the IL-6 -174GC genotype and the remaining 5 patients (6.5%) had the IL-6 -174CC genotype. After stratifying the patients according to IL-6 -174 genotype (Table 5), no significant differences were observed among the subgroups of patients with regard to mean age, ACPA and RF status, disease activity and disability. Patients with the -174CC genotype (C allele) had a significantly lower disease duration compared with those with the GG genotype (*P *= 0.01). A statistically significantly lower MTP TES was observed in patients with the -174GC genotype compared with those with the CC genotype (*P *= 0.007) (Table 5).

**Table 5:** Characteristics of the 77 RA patients stratified by IL-6 -174G/C SNP^a^

Characteristics	GG	GC	CC	*P *value
Mean age, years (± SD)	57.5 ± 13.5	51.1 ± 16.1	61.4 ± 11.9	NS
Mean disease duration, months (± SD)	117.6 ± 74.4	144 ± 129.6	45.6 ± 19.2	*P *= 0.01; GG vs. CC
RF-positive, *n *(%)	39 (81.2)	17 (70.8)	4 (80)	NS
ACPA-positive, *n *(%)	40 (83.3)	16 (66.6)	3 (60)	NS
Mean DAS28 (± SD)	5.3 ± 1.2	4.8 ± 1.3	4.7 ± 0.8	NS
Mean HAQ score (± SD)	1.3 ± 0.7	1.3 ± 0.9	1.3 ± 0.7	NS
Mean overall patient TES (± SD)	36.2 ± 20.4	27 ± 21.8	35.4 ± 28.4	NS
Mean MCP TES (± SD)	14 ± 8.0	12 ± 8.4	12.4 ± 9	NS
Mean PIP TES (± SD)	10.3 ± 7.9	7.5 ± 7.6	13 ± 11.2	NS
Mean MTP TES (± SD)	11.9 ± 7.7	7.4 ± 8.1	10 ± 8.8	*P *= 0.007; GC vs. CC

^a^ACPA: anticitrullinated protein/peptide antibody; DAS28: disease activity score in 28 joints; ESR: erythrocyte sedimentation rate; HAQ: Health Assessment Questionnaire; IL: interleukin; MCP: metacarpophalangeal; MTP: metatarsophalangeal; NS: not significant; PIP: proximal interphalangeal; RA: rheumatoid arthritis; RF: rheumatoid factor; SD: standard deviation; TES: total erosion score.

After subgrouping the patients according to their autoantibody status, no differences were found with respect to genotype. Furthermore, we assessed whether the IL-6-174G/C SNP could have influenced US-identified bone erosion progression during six months of follow-up. After stratifying for the IL-6 -174SNP genotypes, we observed a trend toward a higher rate of progression of bone-erosive damage at T3 and T6 for patients with the -174G allele (GG/GC genotypes).

### Association with response to anti-TNF treatment

After stratifying the patients according to the TGF-β or IL-6 genotype, we observed no significant differences in the proportion of EULAR responders (data not shown). Interestingly, all patients carrying the IL-6 -174CC genotype showed a moderate or good clinical response according to the EULAR criteria after six months of follow-up.

### Interobserver reproducibility

Interobserver agreement was statistically significant with regard to bone erosions (*P *< 0.0001). A comparison of the results from the two sonographers showed that the overall unweighted κ value for the examined joints was 0.72 (agreement in 87.5% of examinations).

## Discussion

In the present study, we have shown that the TGF-β 869C/T SNP could influence the bone-erosive damage evaluated by US in RA patients. Genetic factors are implicated in RA pathogenesis, as they can influence not only RA susceptibility but also clinical and radiological severity and progression [25]. Genetic variants of candidate genes encoding for several cytokines (for example, IL-1, IL-6, IL-10 and TGF-β), proteases (for example, protein tyrosine phosphatase nonreceptor type 22) and other immune/inflammatory genes (for example, macrophage protein 1) have been investigated with contrasting results. When the candidate gene approach was used previously, the study limitations were a lack of statistical power, small cohort dimensions and lack of replication studies [26].

To the best of our knowledge, published data on the effects of polymorphisms on bone-erosive damage in RA have been obtained exclusively on the basis of conventional radiographic assessment [26]. This technique is considered the current standard for the assessment of joint damage in RA [16], but it lacks the capability to detect early bone-erosive damage [27]. Musculoskeletal US is an easily reproducible, time-sparing and relatively low-cost technique that has gained an important role in the evaluation of RA patients. US is increasingly being used in clinical practice related to the standardization of this technique on the basis of the EULAR guidelines and OMERACT definitions [21,23]. US allows an accurate depiction of soft tissues and bony changes at all stages of the disease process. There is a significant correlation between the degree of synovial inflammation as documented by gray scale and power Doppler methods and disease activity indices [28]. Overall, US is more sensitive than radiography in detecting bone erosions in MCP and MTP joints in patients with RA [29-31]. Most of the erosions detected by US cannot be visualized by conventional radiography unless they progress to radiographically evident severe bone lesions, which occur within a period of one to two years. Indeed, US can detect up to seven times more erosions than plain radiography in early RA [32,33].

We evaluated the TGF-β 869C/T and IL-6 -174G/C genetic variants in 77 RA patients with respect to the bone-erosive damage evaluated by US. We chose these SNPs for their potentially key interacting roles in patients with RA. TGF-β is considered a pivotal cytokine in the modulation of the immune response in RA. It shows pro- and anti-inflammatory effects with a broad range of biological functions, including wound healing, fibrosis, immune suppression and angiogenesis [34]. It has chemotactic properties and can stimulate cells to produce IL-1, IL-6, TNF and other proinflammatory cytokines at sites of inflammation [35]. It can have immunosuppressive features by inhibiting the proliferation of T and B cells and the generation of T-cell cytotoxicity [36,37]. The TGF-β 869C/T SNP has been widely evaluated in patients with RA. Studies of Japanese and Chinese patient populations found an association with disease susceptibility not confirmed in Caucasians [11]. A recent meta-analysis underlined that this SNP may play different roles in different ethnicities, as genetic heterogeneity exists in different RA populations [11]. Yamada *et al*. [38,39] found higher TGF-β serum concentrations in patients with CC genotype > > CT genotype and > TT genotype, suggesting that the 869C/T substitution may affect signaling functions of the peptide or the intracellular trafficking or export efficiency of the protein. In our patient population, independently of disease duration, patients with the C allele the number of erosions at the MTP level were almost twice those in patients with the T allele. The C-allele carriers (who are supposed to have higher serum levels of TGF-β) may show increased osteoclast activation mediated by IL-17, leading to erosive damage. Enhanced expression of TGF-β has been detected in synovial effusion and synovium of patients with RA [40]. However, surprisingly, these results were confirmed in ACPA- or RF-positive patients but not in ACPA- or RF-negative patients, in whom opposite results were obtained.

It has been shown that "seropositive" patients display peculiar features, including more severe disease with greater radiographic progression [41]. Our data may support the hypothesis that the autoantibody-positive RA patient population may differ from the so-called "seronegative," not only in clinical outcomes but also with regard to genetic background and mechanism of disease development. The SE allele has been associated with RA only in ACPA-positive patients [42,43]. Because of these considerations, we hypothesize that the dichotomous behavior of TGF-β might be dependent on the presence of autoantibodies that may interact at some level with the TGF-β biologic pathway. In seropositive patients, the presence of a more proinflammatory milieu might switch TGF-β to exert its proinflammatory effects with osteoclastogenic stimulation, leading to bone-erosive damage. In this case, the presence of the C allele might be responsible for the observed higher US-detected bone-erosive damage. On the contrary, in seronegative RA patients, TGF-β might "switch" to exert anti-inflammatory effects, which would explain the lesser bone damage in patients with the C allele. It cannot be excluded that other as yet undiscovered factors may be involved in such a dichotomy. In our study, on the one hand, the inclusion of seropositive and seronegative RA patients led to small subgroup sizes, but on the other hand, this protocol may have helped to clarify the hypothesis that these populations represent different entities, even on a molecular basis. Larger cohorts are required to better address this issue.

Only one previous study investigated the association between TGF-β 869C/T gene SNP and radiographic progression in RA. In agreement with our results, the authors of that study showed that the T allele was not associated with the Larsen score after correction for disease duration. The authors of that publication concluded that the TGF-β 869C/T SNP was not associated with structural severity. According to our results as well, the same SNP is not associated with the progression of structural damage [13].

IL-6 is a proinflammatory cytokine characterized by a range of pleiotropic activities capable of mediating cartilage and bone damage, including induction of acute phase proteins and stimulation of T and B cells, synoviocytes and osteoclasts [44]. The presence of the G variant at position -174 of the promoter region of the IL-6 gene leads to increased transcriptional activity and thus to higher levels of the cytokine in serum and synovial tissue in patients with RA [20,45]. In our study, patients with the -174G allele showed higher rates of progression of erosive damage (although not statistically significant) even in the presence of longer disease duration at baseline. This result is in agreement with the findings of previous studies. Indeed, the same -174G allele was associated with higher disease severity evaluated with the DAS28, and an allele-dose association of the IL-6 -174G variant with increasing radiographic damage was observed in both ACPA-positive and RF-positive RA patients [46].

None of the SNPs in the two genes influence the response to TNF antagonist therapy. This has never been previously addressed for TGF-β 869C/T, while few data showing contrasting results are available for IL-6 -174C/G [47]. As mentioned above, in our study, the MTP joints showed the highest sensitivity of change. It cannot be excluded that statistical significance could have been reached even at the MCP and PIP levels with a larger patient cohort. Nevertheless, forefoot disease activity appears to be frequent in patients with RA. DAS28 score, which excludes forefoot disease from the joint count, may underestimate disease activity compared with DAS44. In a recent study conducted by van der Leeden and colleagues [48], about 40% of the patients who had DAS28 remission had at least one painful and/or swollen MTP joint during the first eight years of RA. These authors suggested that the DAS28 remission criterion for RA neglects patients with active forefoot involvement. In addition to this clinical discrepancy, researchers in several studies have found that the erosive disease in RA often begins in the small joints of the feet, while the hands are affected only at a later stage in the disease course [22]. This evidence is supported by the better sensitivity and specificity in diagnosing RA provided by combining hand and foot radiographs than hand radiographs alone (as per the 1987 ACR criteria) [22,49-51]. More recently, Sheane and colleagues [52] suggested that ultrasonographic assessment of the fifth MTP joints may be useful in the diagnosis of RA by identifying erosions and synovitis at a very early stage. In a previous report published by our group [53], MTP evaluation allowed a distinction to be made between RA and undifferentiated arthritis in patients with early arthritis, suggesting that MTP involvement may be more specific for RA. Thus, evaluation of bone-erosive damage at MTP joints should be performed at an early stage in RA patients.

US has several intrinsic limitations, such as the presence of artefacts produced at the bone cortex, abnormal setting of the machine (that is, increased pulse repetition frequency, reduced gain and altered persistence) and the incapability of penetrating the cortex to identify subchondral lesions, cysts or bone marrow lesions. Nonetheless, the main limits remain spreading and standardization. Despite the great effort being made to expand the use of US, this technique is not yet available to all rheumatologists in the evaluation of RA patients. Moreover, there is a certain level of inter- and intraobserver variability between ultrasonographers.

However, in the evaluation of bone erosions, US has demonstrated good interobserver agreement in studies reported in the literature [54]. In our study, to reduce operator dependence to a minimum, the patients were studied independently on the same day by two experienced ultrasonographers. The results of the erosion scores were the means of the scores recorded by the two operators, and the interobserver agreement was high and comparable to that reported in previous studies.

## Conclusions

In conclusion, our study confirms that genetic factors are involved in determining the severity of bone damage in RA as well as in predicting disease progression. Of note, the TGF-β 869C/T SNP seems to have dichotomous roles according to the patient's autoantibody (ACPA and RF) status. To the best of our knowledge, this study is the first in which the roles of TGF-β and IL-6 gene variants in bone-erosive damage were evaluated with US. Further studies with larger patient series and longer follow-up are needed.

## Abbreviations

ACPA: anticitrullinated protein/peptide antibody; CRP: C-reactive protein; DAS28: disease activity score in 28 joints; ELISA: enzyme-linked immunosorbent assay; ESR: erythrocyte sedimentation rate; EULAR: European League Against Rheumatism; HAQ: Health Assessment Questionnaire; HLA: human leukocyte antigen; IL: interleukin; MCP: metacarpophalangeal; MTP: metatarsophalangeal; OMERACT: Outcome Measures in Rheumatoid Arthritis Clinical Trials; PCR: polymerase chain reaction; PIP: proximal interphalangeal; RA: rheumatoid arthritis; RF: rheumatoid factor; SD: standard deviation; SE: shared epitope; SNP: single-nucleotide polymorphism; TES: total erosion score; TGF-β: transforming growth factorβ; TNF: tumor necrosis factor; US: musculoskeletal ultrasound; VAS: visual analogue scale.

## Competing interests

The authors declare that they have no competing interests.

## Authors' contributions

FC designed the study, conducted the clinical evaluation, performed the ultrasonographic assessment and drafted the manuscript. CP was involved in the design of the study and performed the ultrasonographic assessment and the statistical analysis. MF carried out the molecular genetic studies and drafted the manuscript. CA and AI were involved in the design and conception of the study and helped with drafting the manuscript. CF and EP carried out the molecular genetic studies. SDV and GV were involved in the design of the study and helped with drafting the manuscript. All authors read and approved the final manuscript.
